# Impact of Vaccination and Prior Infection on SARS-CoV-2 Viral Load in Preschool Children During the Omicron Pandemic

**DOI:** 10.3390/vaccines13080850

**Published:** 2025-08-11

**Authors:** Mitsuyoshi Suzuki, Akifumi Tokita, Mariko Inaba, Yoshimi Tada, Kyoko Shuri, Asako Miura, Mitsuharu Fukazawa, Masashi Fujioka, Yuko Sakai-Tagawa, Seiya Yamayoshi, Kiyoko Iwatsuki-Horimoto, Yoshihiro Kawaoka, Masaaki Miyazawa

**Affiliations:** 1 Department of Pediatrics, Juntendo University Faculty of Medicine, 2-1-1 Hongo, Bunkyo-ku, Tokyo 113-8421, Japan; a.tokita@bambini.tokyo (A.T.); m.otsuka.fg@juntendo.ac.jp (M.I.); 2 Clinic Bambini, 3-16-13 Shirokanedai, Minato-ku, Tokyo 108-0071, Japan; 3 Shibaura Clinic, 4-20-4 Shibaura, Minato-ku, Tokyo 108-0023, Japan; tada@shibaurakodomo.com; 4 Sunny Garden Clinic Pediatrics, 2-16-4 Azabujyuban, Minato-ku, Tokyo 106-0045, Japan; hopeyoursmile@sunnygardencl.com; 5 Polepole Clinic, 1-5-21 Takanawa, Minato-ku, Tokyo 108-0074, Japan; miura@polepole-clinic.com; 6 Fukazawa Children’s Clinic, 3-2-33 Wakamiya, Higashi-ku, Fukuoka 813-0036, Japan; mitsuharu0720@yahoo.co.jp; 7 Fujioka Pediatric Clinic, 2-15-16 Kunokidai, Tondabayashi-shi, Osaka 584-0074, Japan; fjok@silver.ocn.ne.jp; 8 Division of Virology, Institute of Medical Science, The University of Tokyo, 4-6-1 Shirokanedai, Minato-ku, Tokyo 108-8639, Japan; ytsakai@g.ecc.u-tokyo.ac.jp (Y.S.-T.); yamayo@ims.u-tokyo.ac.jp (S.Y.); kenken@ims.u-tokyo.ac.jp (K.I.-H.); yoshihiro.kawaoka@wisc.edu (Y.K.); 9 The University of Tokyo Pandemic Preparedness Infection and Advanced Research Center (UTOPIA), The University of Tokyo, Minato-ku, Tokyo 108-8639, Japan; 10 International Virus Infectious Disease Research Center, National Institute of Global Health and Medicine, Japan Institute for Health Security, Shinjuku-ku, Tokyo 162-8655, Japan; 11 Department of Special Pathogens, International Research Center for Infectious Diseases, Institute of Medical Science, The University of Tokyo, Minato-ku, Tokyo 108-8639, Japan; 12 Influenza Research Institute, Department of Pathobiological Sciences, School of Veterinary Medicine, University of Wisconsin-Madison, 575 Science Drive, Madison, WI 53711, USA; 13 Research and Development Center for Nasal Mucosal Vaccine, Translational Research Business Division, Shin Nippon Biomedical Laboratories, 2438 Miyanoura-cho, Kagoshima-shi, Kagoshima 891-1394, Japan; 14 Kindai University, 3-4-1 Kowakae, Higashi-Osaka-shi, Osaka 577-8502, Japan

**Keywords:** COVID-19, rapid antigen tests, pre-school children, vaccination, viral load

## Abstract

**Background:** Preschool-aged children can have difficulty adhering to infection control measures and were affected during the Omicron wave of the coronavirus disease 2019 (COVID-19) pandemic. However, the impacts of prior severe acute respiratory syndrome coronavirus 2 (SARS-CoV-2) infection and vaccination on viral load in this age group remain poorly understood. This study aimed to investigate the relationship between previous SARS-CoV-2 infection, COVID-19 vaccination, and viral load or clinical severity in preschool-aged children infected during the Omicron variant epidemic in Japan. **Methods:** This prospective observational study investigated 107 children aged 1–75 months who were diagnosed with COVID-19 between May and September 2023. Rapid antigen (Ag) tests were performed on days 1 and 5 or 6, and results were visually graded into four categories (–, ±, 1+, or 2+). Ag results were validated against quantitative real-time reverse transcription polymerase chain reaction (RT-qPCR) cycle threshold (Ct) values. Clinical parameters, including vaccination status, previous infection, age, maximum body temperature, and fever duration, were analyzed using multivariate regression models. **Results:** Higher Ag loads (1+/2+) were more frequently observed in younger children who had not experienced prior infection or full vaccination. Prior infection and vaccination were independently linked to lower Ag loads and reduced maximum body temperature. Many unvaccinated and infection-naïve children continued to show elevated Ag levels on day 5 or 6, corresponding to Ct values suggestive of potential infectivity. **Conclusions:** Prior SARS-CoV-2 infection and vaccination were linked to lower viral loads and milder febrile responses among preschool-aged children. These findings enhance our understanding of infection dynamics in this age group and may inform future discussions on public health strategies in pediatric settings.

## 1. Introduction

The coronavirus disease 2019 (COVID-19) pandemic, caused by severe acute respiratory syndrome coronavirus 2 (SARS-CoV-2), has significantly impacted global health, economies, and daily life since its emergence in late 2019 [1]. Although initially thought to predominantly affect adults, with generally mild cases reported among children [2], the situation evolved with the emergence of the Omicron variant and its sublineages, which showed increased transmissibility, including among younger, previously less-affected populations such as preschool-aged children [3].

The use of face masks by children aged two and older in childcare settings reduced COVID-19-related program closures over the course of a year, highlighting their role in supporting safe, continuous care and informing public health policies [4]. However, preschool-aged children represent unique challenges for infection control due to their limited ability to consistently adhere to public health measures such as mask-wearing, physical distancing, and hand hygiene. Adherence to mask use among young children tends to decline over time, and practical implementation often requires substantial support from parents or caregivers [5]. Behaviorally, adherence to infection control measures like mask-wearing is known to be limited in young children due to developmental constraints [4]. In addition, the World Health Organization advises against mask usage for asymptomatic children aged 2–5, citing concerns about their ability to wear face masks properly without supervision, and the potential negative impacts on learning and psychosocial development [6]. Immunologically, both type I interferon (IFN-I) responses and the production of neutralizing antibodies are earlier and stronger, and lymphopenia is rarely observed in young children upon SARS-CoV-2 infection. Further, T-cell receptor specificity and cytokine production profiles after SARS-CoV-2 infection differ between children and adults, probably associated with the above-mentioned generally mild symptoms. However, some childhood-onset genetic conditions can be related to IFN-I autoantibodies and defects in its signal transduction pathways, which can lead to severe COVID-19 [7].

Despite typically experiencing milder disease symptoms, children remain at risk for complications such as multisystem inflammatory syndrome in children and long COVID [8]. In Japan, the dynamics of SARS-CoV-2 infection among children have shown some unique features. During the Omicron wave, large surges of pediatric cases were reported, with frequent transmission clusters identified in nursery schools and kindergartens [9]). The mRNA vaccine (Pfizer-BioNTech, Tokyo, Japan) received approval for administration to children aged 6 months to 5 years relatively late, on 24 October 2022, after significant waves of community transmission had already taken place. As a result, many young children remained unvaccinated or received incomplete immunizations during the peak periods of Omicron transmission.

Vaccination has proven effective in reducing severe disease and viral load in adult age groups [10]. However, real-world data on the impact of vaccination on viral kinetics in young children remains limited. A recent study in the United Kingdom showed that vaccination was associated with a reduced risk of infection and milder disease among children and adolescents aged 12–17 years during the Omicron era [11]. Nevertheless, evaluations among younger children who are either vaccine-naïve or have only recently been immunized are still scarce. Further, on 8 May 2023, Japan’s Ministry of Health, Labour and Welfare reclassified COVID-19 from a Category 2 to a Category 5 infectious disease, aligning its legal handling with that of seasonal influenza. Current guidelines recommend a minimum of 5 days of self-isolation after symptom onset. However, these recommendations are primarily based on adult data and may not accurately reflect viral shedding and infectivity timelines in young children [12,13]. In Japan, the COVID-19 vaccination rate for children is notably low, with considerable regional differences. Optimization of public health guidelines, therefore, requires determination of differences in clinical severity and duration of infectious virus shedding based on vaccination history or previous infection.

This study aimed to address these gaps by evaluating the relationship between vaccination history, prior SARS-CoV-2 infection, and viral load in preschool-aged children diagnosed with COVID-19 during the Omicron wave in Japan. By utilizing rapid antigen (Ag) tests validated against quantitative real-time reverse transcription polymerase chain reaction (RT-qPCR) assays, this investigation sought to inform isolation guidelines and public health measures based on local, pediatric-specific data.

## 2. Methods

### 2.1. Cycle Threshold (Ct)-Based Validation of Visual Ag Test Grading

Prior to this study, rapid Ag test results were compared with corresponding RT-qPCR Ct values from a total of 227 samples collected from children under 15 years of age between January 2021 and February 2023 to validate the semi-quantitative Ag load categorization against viral load. Ct values exceeding 30 were established as indicative of low infectious potential based on previous virus culture studies [14,15]. Initial validation with a smaller number of cases without clinical analyses has been reported in Japanese [16].

**Figure 1:**
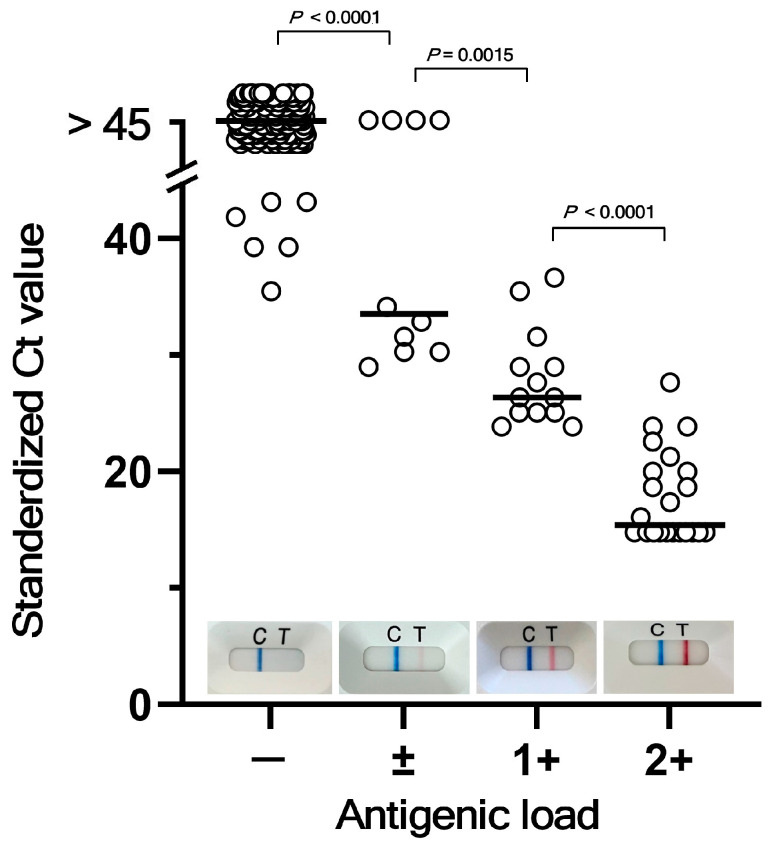
Correlation between quasi-quantitative Ag test results and RT-qPCR Ct values. Rapid Ag test results were visually categorized as negative (−), faint positive (±), moderate positive (1+), and strong positive (2+) as shown by representative photographs. Horizontal bars indicate mean Ct values for each Ag test category. As Ct values did not pass the Kolmogorov–Smirnov normal distribution test for any of the four Ag load groups except the 1+ category, one-way ANOVA using the Kruskal–Wallis test was performed and revealed group-wise differences (*p* < 0.0001). The two-tailed Mann-Whitney test indicated significant group-wise differences, as shown with corresponding *p* values.

A COVID-19 diagnosis was confirmed using a qualitative rapid antigen test (QuickNavi™-COVID19 Ag; Denka Co., Tokyo, Japan) performed on nasopharyngeal swabs at initial presentation [17]. Rapid Ag test results were visually categorized as: negative (–), defined by no positive line visible at the time of judgment; faint positive (±), with a faint line visible at the time of judgment; moderate positive (1+), with a fully developed positive line observed by the predetermined time of judgment; and strong positive (2+), with a positive line appearing within 30 s after sample dropping. Representative photographs are shown for these categories in Figure 1. To ensure inter-observer reproducibility, the categorization was conducted by two independent, trained healthcare providers (physicians or clinical nurses).

RT-qPCR assays were performed using the Smart Gene^®^ SARS-CoV-2 RNA detection reagent (Mizuho Medy Co., Saga, Japan), an automated molecular testing platform that employs the quenching probe method [18]. For RT-qPCR–positive cases, Ct values were standardized using a conversion formula previously by validated Kiyasu et al.: X = (Y − 11.497)/0.7757, where X represents the Ct value standardized according to the National Institute of Infectious Diseases (NIID), Japan, and Y denotes the Smart Gene cycle number [18]. This standardization was necessary to ensure comparability of Ct values obtained from the Smart Gene^®^ platform with reference values established by the NIID. The conversion allowed uniform interpretation of viral load across samples analyzed on different platforms.

### 2.2. Study Design and Patient Enrollment

This prospective observational study included 107 preschool children aged 1 to 75 months (median age, 36 months), diagnosed with COVID-19 using the above rapid qualitative Ag test at participating pediatric clinics from 1 May to 30 September 2023. Eligible participants displaying symptoms such as fever were identified as close contacts of confirmed COVID-19 cases or were involved in localized community outbreaks. Participants were prospectively recruited from outpatient pediatric clinics in Tokyo, Osaka, and Fukuoka, Japan. Enrollment was based on symptom presentation or contact history, and written informed consent was obtained from guardians. Rapid Ag testing was reperformed on day 5 or 6 after symptom onset. Clinical data collected during the initial and follow-up visits (day 5 or 6 after symptom onset) included age, sex, vaccination status (number of doses and time elapsed since the last dose), history of prior SARS-CoV-2 infection, maximum body temperature, and duration of fever.

In this study, all vaccinated participants received the Pfizer-BioNTech mRNA vaccine (BNT162b2). Only the third dose given to Case No.81 (Supplementary Table S1) was the bivalent formulation targeting both the ancestral strain (Wuhan-Hu-1) and Omicron BA.4/5; while all other doses were of the original monovalent vaccine targeting the ancestral strain. Any missing data were collected later through telephone interviews with guardians. Concurrent with the initial antigen test, variant analysis for SARS-CoV-2 was performed on nasopharyngeal swabs from several affected children. Supplementary Table S1 summarizes the demographic and clinical data, as well as the vaccination and infection histories, of the participating children.

During the study period, we collected nasopharyngeal swabs from 15 children (15/107 cases) to identify SARS-CoV-2 variants. These samples were then analyzed using whole-genome sequencing or variant-specific typing to determine the clades and lineages of circulating SARS-CoV-2 (Table 1). Sequencing analysis was performed using the Illumina platform, and variant classification adhered to the standard Nextclade_Pango nomenclature, as previously described [19]. All experiments with SARS-CoV-2 were conducted in enhanced biosafety level 3 (BSL-3) containment laboratories at The University of Tokyo.

### 2.3. Ethical Considerations

The study was conducted in accordance with the Declaration of Helsinki and approved by the institutional review board of the Institute of Medical Science, The University of Tokyo (approval no. 2024-76-0116, recognition date 16 January 2025), and Juntendo University Faculty of Medicine (approval no. E22-0281, recognition date 9 September 2022; revised, approval no. C24-0166, recognition date 19 February 2025).

### 2.4. Statistical Analysis

Descriptive statistics were summarized as means ± standard deviation or median with interquartile ranges (IQR). The normality of data distributions was assessed using the Normality and Lognormality Tests function of GraphPad Prism software (version 10.5.0; GraphPad Software, Boston, MA, USA). Comparative analyses utilized the Mann–Whitney U tests for continuous variables and Fisher’s exact tests or the chi-squared tests for categorical variables. Multivariate logistic regression analysis identified independent factors associated with higher Ag loads (categories 1+ and 2+). Multiple linear regression analyses assessed the effects of age, prior infections, and vaccination on viral load and clinical severity (reflected by maximum body temperature). A two-tailed *p*-value less than 0.05 was considered statistically significant. Graphs were created with the GraphPad Prism software, and detailed methods of statistical analyses utilized are described in each corresponding figure legend. Sensitivity, specificity, positive and negative predictive values and their confidence intervals (CI) were calculated by using the Diagnostic test evaluation calculator provided by MedCalc Software Ltd. (Ostend, Belgium) at https://www.medcalc.org/calc/diagnostic_test.php (accessed on 2 August 2025).

## 3. Results

### 3.1. Correlation Between Quasi-Quantitative Ag Load Analyses and RT-qPCR for SARS-CoV-2

To validate Ag load categorization, rapid Ag test results from 227 samples were plotted against the corresponding RT-qPCR Ct values (Figure 1). Ct value distributions showed significant differences among the four Ag categories, confirming the semi-quantitative nature of the Ag categorization. Importantly, all samples categorized as 2+ exhibited Ct values below 30, indicating potentially infectious viral loads. When Ag test positivity was defined as ±, 1+, or 2+ with Ct ≤ 30 as the infectivity threshold, the sensitivity was 100% (95% CI 89.4–100%), specificity was 93.8% (95% CI 89.4–96.8%), positive predictive value (PPV) was 78% (95% CI 61.4–82.6%), and negative predictive value (NPV) was 100% (95% CI 89.4–100%). On the other hand, when Ag test positivity was defined as 1+ or 2+, the sensitivity was 97% (95% CI 84.2–99.9%), specificity was 98.5% (95% CI 95.6–99.7%), PPV was 91.4% (95% CI 77.6–97.0%), and NPV was 99.5% (95% CI 96.5–99.9%). These figures indicate that defining Ag positivity with 1+ and 2+ results in improved prediction of potential infectivity without sacrificing sensitivity. These findings also suggest that the Ag test, with its high sensitivity and NPV, is practical for ruling out infectivity in pediatric settings.

### 3.2. Analysis of Factors Associated with Viral Load Mitigation

#### 3.2.1. Demographic and Immunization Characteristics of the Study Cohort

Among the 107 preschool children enrolled, the mean age was 36 ± 21.6 months, with a mean maximum body temperature of 39.0 ± 0.74 °C and a mean fever duration of 2.1 ± 0.91 days. Nineteen children (17.8%) had received at least two vaccine doses (15 had three doses, 4 had two doses), and the mean interval since their last vaccination was 5.9 ± 1.9 months (Supplementary Table S1).

#### 3.2.2. SARS-CoV-2 Variant Analysis

Genetic analysis to identify SARS-CoV-2 variants was conducted on samples from 15 consenting participants. The identified variants are summarized in Table 1. This variant-tested subgroup was distinct from those used for RT-PCR validation analyses. The purpose of this analysis was to determine the predominant circulating strain during the study period, confirming that Omicron and its subvariant XBB dominated this period.

#### 3.2.3. Factors Influencing Viral Load at the End of the Isolation Period

Ag loads on days 5 or 6 after symptom onset (marking the end of the recommended isolation period) were categorized into high (1+ and 2+; *n* = 58) and low viral load (– and ±; *n* = 49) groups. Univariate analyses revealed that older age, prior vaccination (≥2 doses), and previous SARS-CoV-2 infection were significantly associated with lower viral loads (Table 2).

#### 3.2.4. Relationship Between Ag Load, Vaccination, and Prior Infection

**Figure 2:**
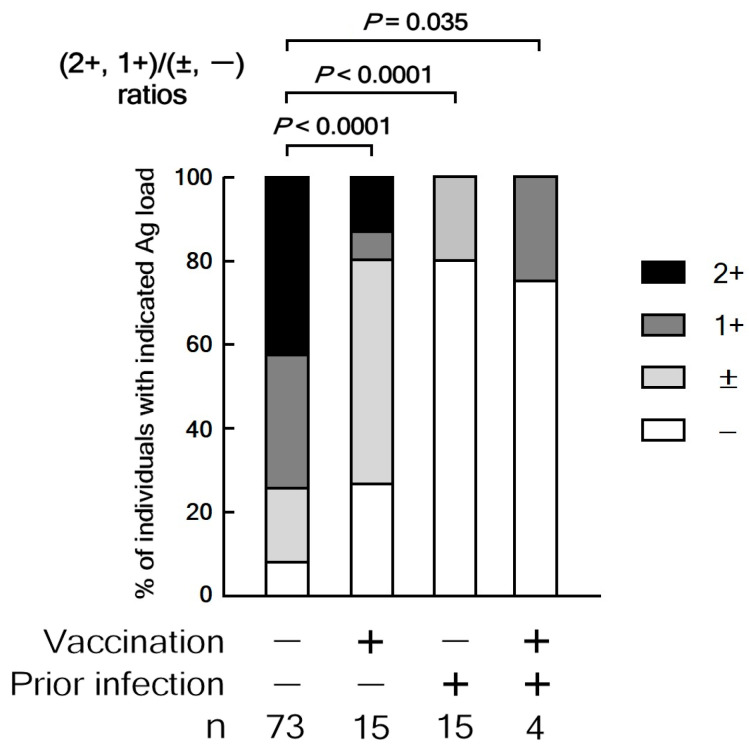
Distribution of Ag load categories stratified by prior infection and vaccination status. The proportions of Ag load categories (–, ±, 1+, and 2+) on day 5 or 6 after symptom onset are presented according to participants’ prior SARS-CoV-2 infection and vaccination history. Significantly different distribution of Ag load categories between prior infection and vaccination status groups is shown by the extended Fisher’s exact probability test on 2 by 4 contingency tables of raw numbers. Two-tailed Chi-square tests were then performed on ratios of high (1+ or 2+) and low (– or ±) Ag load individuals.

Figure 2 shows the Ag load category distributions, stratified by prior infection and vaccination history. Extended Fisher’s exact tests on 2 by 4 contingency tables showed significant differences in the distribution of four Ag load categories between the unvaccinated and infection-naïve group and three other groups of children (*p* < 0.0001 to *p* = 0.0029). Therefore, ratios of the number of individuals with high (1+ or 2+) and low (− or ±) Ag load categories were compared between the unvaccinated and infection-naïve group and each of the three other groups. Children who get SARS-CoV-2 infection without prior infection or vaccination exhibit a significantly higher frequency of high Ag load levels compared to those who have had previous infection or vaccination, or both. These findings indicate that prior infection or vaccination reduces the Ag load 5 or 6 days after symptoms start in preschool-aged children.

#### 3.2.5. Multivariate Analysis of Factors Associated with High Antigen Load

To further assess the independent contributions of these factors, we performed logistic regression analysis using Ag load positivity (1+ or 2+) as the dependent variable. As shown in the logistic regression model (*n* = 107; area under the receiver operating characteristic curve, 0.833), both prior infection and vaccination were significantly associated with a lower likelihood of high Ag load. Specifically, prior infection was associated with a markedly reduced likelihood of an elevated Ag load (β = −3.251; odds ratio [OR] 0.039 95% confidence interval [CI] 0.005–0.323; *p* = 0.0027), and vaccination was also associated with a reduced risk (β = −1.944; OR 0.143, 95%CI 0.040–0.508; *p* = 0.0026). Age in months was not significantly associated with Ag load (β = −0.021; OR 0.979, 95%CI 0.957–1.002; *p* = 0.072), suggesting no clear relationship between age and antigen load in this cohort. These findings indicate that both prior infection and vaccination independently contribute to lowering viral antigen load in preschool children with SARS-CoV-2, reinforcing their role in modulating disease severity during the Omicron pandemic.

#### 3.2.6. Impact of Prior Infection and Vaccination on Clinical Severity

**Figure 3:**
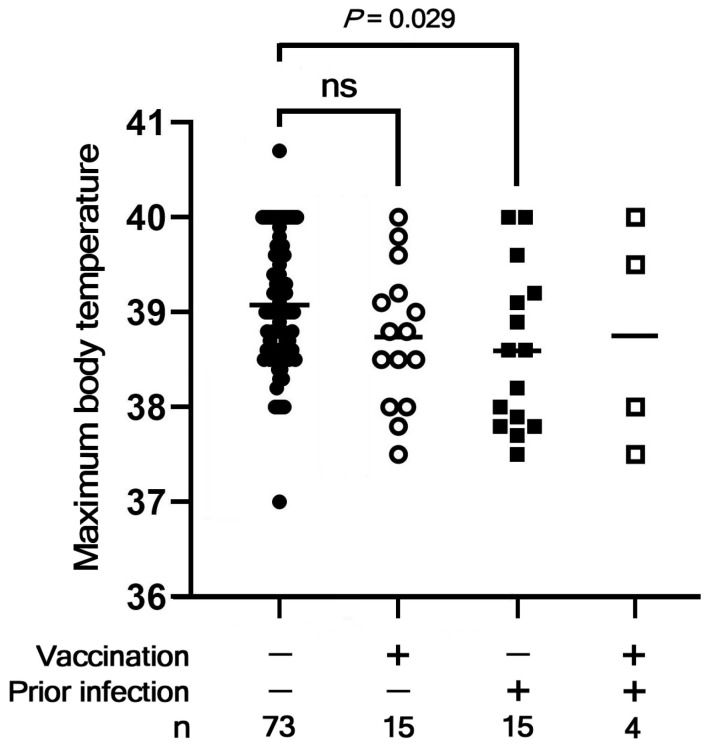
Maximum body temperature stratified by infection and vaccination status. Maximum body temperatures recorded during illness are presented according to participants’ prior infection and vaccination status. As the distribution of body temperature values for the unvaccinated and infection-naïve group did not pass the Kolmogorov–Smirnov normal distribution test, a two-tailed Mann-Whitney test was used to examine possible group-wise differences.

Multiple linear regression analysis revealed that maximum body temperature was significantly lower in children with prior SARS-CoV-2 infection (estimated coefficient: −0.541, 95%CI −0.974 to −0.107; *p* = 0.0150). Fever duration, however, did not show a significant correlation with infection history. Vaccinated children exhibited a trend toward lower maximum body temperature, although this was not significant, while children with prior SARS-CoV-2 infection exhibited significantly lower maximum body temperature in comparison with those without prior infection or vaccination (Figure 3). These different effects of previous infection and vaccination may be due to the limited number of fully vaccinated children in our cohort, which could have led to insufficient statistical power to detect moderate differences. It is important to note that factors such as age, sex, and underlying health conditions might also affect clinical severity and were not fully controlled for in this study. These results, however, suggest that acquired immunity (either from previous infection or vaccination) may lessen clinical severity in preschool-aged children.

## 4. Discussion

Our findings show that both prior SARS-CoV-2 infection and COVID-19 vaccination significantly lower viral load and maximum body temperature in preschool-aged children who were infected during the Omicron variant epidemic. These results align with previous studies in adults and adolescents [20,21], while expanding the evidence to the previously underrepresented population of children under five years old. This highlights the critical necessity to expand vaccine coverage and enhance epidemiologic surveillance in this at-risk age group.

Reducing viral load has significant implications not only for individual clinical outcomes but also for public health, particularly in high-contact environments such as preschools and childcare centers. Our data indicated that a substantial proportion of unvaccinated and infection-naïve preschoolers continued to show high Ag loads (classified as 1+ or 2+) on days 5 or 6 after symptom onset. These Ag intensities corresponded to RT-qPCR Ct values below 30, a threshold associated with potential infectivity [14,15]. This is particularly concerning given young children’s limited ability to consistently adhere to preventive measures like mask-wearing and physical distancing [2,3]. Accordingly, the current fixed 5-day isolation policy in Japan may be insufficient for this age group. A fact-based strategy using rapid Ag tests may offer a more individualized and evidence-driven approach to determining safe return-to-care timelines.

The semi-quantitative visual interpretation of Ag test results utilized in this study provides a practical and straightforward method for estimating viral load, particularly in resource-limited or primary care settings where the ability to perform PCR may be restricted. Our validation analyses confirmed a strong inverse correlation between Ag signal intensity and RT-qPCR Ct values, consistent with previous reports linking higher Ag positivity to greater infectivity [14]. These findings support the use of rapid Ag testing not only as a diagnostic tool but also as an indicator of infectiousness in pediatric populations. Furthermore, when Ag positivity was defined as visual categories of 1+ or 2+, the rapid antigen test showed a sensitivity of 97%, specificity of 98.5%, PPV of 91.4%, and NPV of 99.5% in detecting cases with adjusted Ct values ≤ 30. These metrics indicate that a positive Ag result consistently detects children with high viral loads and infectiousness, while a negative result strongly suggests a low transmission risk. This high diagnostic accuracy highlights the usefulness of rapid Ag testing not only for initial diagnosis but also for guiding test-based isolation plans and determining when it is safe for children to return to care in preschool settings.

Interestingly, while both vaccination and prior infection were associated with lower viral loads, only prior infection was significantly associated with reduced maximum body temperature in the present study. This may reflect the broader and more diverse immune responses elicited by natural infection compared to the narrower spike protein–focused immunity generated by current vaccines, particularly in younger children [7,22]. Natural infection can trigger both mucosal and systemic immune responses, providing broader protection, while current mRNA vaccines mainly induce systemic immunity focused on the spike protein [23]. These immunologic differences may explain the differing impacts observed on fever response and viral clearance. In this study, all vaccinated participants received the mRNA-based Pfizer-BioNTech vaccine. Although the impact of vaccine type could not be assessed, future research comparing different vaccine platforms (e.g., inactivated or protein subunit vaccines) may provide further insights into the effects on viral kinetics in children.

Recent reports have noted an increase in febrile seizures and encephalopathy among children in East Asia, including Japan, during the COVID-19 pandemic. Notably, the rate of febrile seizures rose during the Omicron variant wave, with a higher prevalence of complex febrile seizures and status epilepticus [24,25]. Additional studies have highlighted a rise in central nervous system abnormalities related to COVID-19, such as seizures, altered consciousness, and suspected acute encephalopathy, particularly in preschool and elementary-school-aged children. Moreover, cases of status epilepticus–type acute encephalopathy associated with COVID-19 have also been reported, underscoring the importance of early medical intervention [26]. Findings from these cohort studies have suggested that prior immunity, particularly when derived from both infection and vaccination, may accelerate viral clearance, mitigate clinical severity, reduce the risk of transmission, and potentially help prevent febrile seizures and neurologic complications.

When pregnant or breastfeeding mothers receive the COVID-19 vaccine, the antibodies produced are transferred to their babies through the placenta and breast milk, which is expected to help protect the babies from infection. Increased levels of anti-SARS-CoV-2 immunoglobulin (Ig)A and IgG antibodies have been observed in breast milk of mothers recovered from COVID-19 [27]. In this study, no differences in viral loads were observed on days 5 or 6 after symptom onset, regardless of variations in nutritional practices from newborn to infancy period. While it is unclear whether mothers in this study had prior infections, this finding may have been influenced by the fact that vaccination rates were lower and equivalent between children and mothers, and that most participants had already completed breastfeeding. However, the effectiveness and duration of this maternal-derived protection in preschool-aged children remain unclear, and further research is warranted to elucidate its role.

This study showed several limitations. First, the number of fully vaccinated participants was relatively small (*n* = 19), limiting the statistical power for assessing vaccine effectiveness in a robust manner. This reflects broader national trends, in which vaccine uptake among young children remains low due to ongoing parental concerns regarding its safety, efficacy, and necessity. Second, the lack of antibody titer measurements limited our ability to correlate immune status with viral load and clinical outcomes. Third, the observational nature of the study prevented reaching any definitive conclusions about causality. Fourth, whole-genome sequencing and variant typing were performed in only a subset of participants, restricting the generalizability of variant-specific findings. Finally, the study did not include detailed immunologic assessments, such as antibody titers or T-cell responses, which could have provided deeper insights into the mechanisms of immune protection. Future studies incorporating viral culture, comprehensive immunologic profiling, and longitudinal follow-up will be essential to fully elucidate the immune dynamics and infectivity in this age group.

## 5. Conclusions

In conclusion, our data provide compelling evidence that prior SARS-CoV-2 infection and vaccination reduce viral load and peak body temperature in preschool-aged children upon infection with Omicron or its subvariants. These findings support considering updated isolation guidelines that include test-based criteria for safe reintegration into group settings. Additional studies with larger vaccinated cohorts are necessary to validate these results. Furthermore, our findings highlight the urgent need to advocate for pediatric vaccination and develop evidence-based policies specifically designed for the preschool age group in light of evolving viral variants and public health demands.

## Figures and Tables

**Table 1:** SARS-CoV-2 variants identified among a subset of participants.

Date of Specimen Collection	Age (Mon)	Sex	Clade	Nextclade Pango Lineage	Alias Designation
2023-05	11	M	22F (Omicron)	XBB.1.4	XBB.1.4
2023-05	49	F	22D (Omicron)	BN.1.2	BA.2.75.5.1.2
2023-07	22	F	23A (XBB.1.5)	XBB.1.5	XBB.1.5
2023-07	5	F	22F (XBB)	XBB.1.33	XBB.1.33
2023-07	1	M	22F (XBB)	XBB.1.33	XBB.1.33
2023-08	54	M	23F (EG.5.1)	EG.5.1	XBB.1.9.2.5.1
2023-08	9	F	23D (XBB.1.9)	XBB.1.9.2	XBB.1.9.2
2023-08	19	M	23F (EG.5.1)	EG.5.1.1	XBB.1.9.2.5.1.1
2023-08	41	M	23F (EG.5.1)	EG.5.1.	XBB.1.9.2.5.1
2023-08	16	M	23A (XBB.1.5)	GK.1.1	XBB.1.5.70.1.1
2023-08	37	F	23C (CH.1.1)	JL.1	BA.2.75.3.4.1.1.1.1.17.1.3.2.1
2023-08	68	F	23C (CH.1.1)	JL.1	BA.2.75.3.4.1.1.1.1.17.1.3.2.1
2023-08	26	F	23F (EG.5.1)	EG.5.1	XBB.1.9.2.5.1
2023-08	5	F	23D (XBB.1.9)	XBB.1.9.2	XBB.1.9.2
2023-08	1	F	23E (XBB.2.3)	XBB.2.3.5	XBB.2.3.5

Whole-genome sequencing or variant-specific typing was performed on nasopharyngeal swab specimens from 15 participants. For each sample, the clade, Nextclade Pango lineage, and alias designation are provided. Mon: months; Sex: F—female, M—male.

**Table 2:** Comparison of clinical and epidemiological characteristics between children with High and low viral loads on day 5 or 6 after symptom onset.

Variable	High Ag Load *n* = 58	Low Ag Load *n* = 49	*p*-Value
Female, *n* (%)	26 (44.8%)	25 (51.0%)	0.59 ^c^
Age (months), median (IQR)	30 (11–47)	45 (28–60)	<0.001 ^a^
Infant nutrition method			
Breastfed, *n* (%)	22 (37.9%)	18 (36.7%)	0.75 ^c^
Vaccination status			
≥2 doses, *n* (%)	3 (5.2%)	13 (27.1%)	0.005 ^b^
History of prior infection, *n* (%)	1 (1.7%)	18 (37.5%)	<0.001 ^b^

Children were divided into two groups based on their antigen test results on Day 5 or 6 after symptom onset: the High Ag Load group with visual categories of 1+ or 2+, and the Low Ag Load group with categories of ± or −. Statistical analyses: ^a^ Mann–Whitney U test; ^b^ Fisher’s exact test; ^c^ Chi-squared test. Fisher’s exact test was used instead of the chi-squared test because some subgroups had only a small number of individuals (*n* < 5 in certain cells).

## Data Availability

The original contributions presented in this study are included in the article/Supplementary Material. Further inquiries can be directed to the corresponding authors.

## Supplementary Materials

The following supporting information can be downloaded at: https://www.mdpi.com/article/10.3390/vaccines13080850/s1, Table S1: Clinical characteristics and antigen test results of preschool children enrolled in the study.
