# Supplementary material for: Impact of Vaccination and Prior Infection on SARS-CoV-2 Viral Load in Preschool Children During the Omicron Pandemic
Source: Vaccines (Basel). 2025 Aug 11;13(8):850. doi: 10.3390/vaccines13080850 (PMC12390252; doi:10.3390/vaccines13080850)
Supplement: Supplementary file 1 [file vaccines-13-00850-s001.zip › vaccines-3753900-supplementary.pdf]

Supplemental Table S1. Clinical characteristics and antigen test results of preschool children enrolled in the study

Case	Infection type 1: primary 2: secondary	Age (months)	Sex	Infant nutrition method	Vaccination count	Interval since last vaccination (months)	Maximum fever (°C)	Fever duration (days)	Antigen test result (day 5 or 6)
1	1	1	M	B	0		38.5	3	2+
2	1	1	F	B	0		38.0	2	2+
3	1	1	F	B	0		38.3	2	2+
4	1	4	M	MF	0		39.2	3	2+
5	1	5	F	B	0		38.5	1	2+
6	1	8	M	MF	0		38.8	3	2+
7	1	9	M	B	0		37.0	0	2+
8	1	11	M	MF	0		39.5	3	2+
9	1	16	F	MF	0		38.6	1	2+
10	1	13	M	MF	0		40.7	3	2+
11	1	22	M	MF	0		39.4	3	2+
12	1	12	M	B	0		40.0	3	2+
13	1	21	F	MF	3	8	37.8	2	2+
14	1	26	M	MF	0		39.0	2	2+
15	1	25	F	B	0		39.2	2	2+
16	1	42	M	MF	0		39	2	2+
17	1	38	F	MF	0		38.2	4	2+
18	1	41	M	A	0		39.0	3	2+
19	1	40	F	B	0		38.0	1	2+
20	1	44	F	MF	0		39.7	3	2+
21	1	36	M	MF	0		39.4	4	2+
22	1	49	F	MF	0		40.0	3	2+
23	1	59	F	B	0		39.0	2	2+
24	1	66	M	A	0		40.0	2	2+
25	1	71	F	B	0		38.9	2	2+
26	1	2	M	MF	0		38.3	1	2+
27	1	3	M	MF	0		39.1	1	2+
28	1	10	F	B	0		38.7	3	2+
29	1	11	M	MF	0		38.0	2	2+
30	1	22	M	A	0		39.0	3	2+
31	1	16	F	B	0		40.0	2	2+
32	1	32	F	MF	3	5	38.8	4	2+
33	1	64	F	B	0		40.0	3	2+
34	1	6	M	B	0		39.0	2	1+
35	1	6	M	B	0		38.5	1	1+
36	1	8	M	A	0		38.4	4	1+
37	1	9	F	B	0		39.6	4	1+
38	1	14	F	B	0		39.3	2	1+
39	1	18	F	MF	0		39.0	2	1+

40	1	22	F	MF	0		38.6	2	1+
41	1	28	F	MF	0		40	1	1+
42	1	30	M	MF	0		39.6	2	1+
43	1	42	M	A	0		39.6	1	1+
44	1	50	M	B	0		40.0	3	1+
45	1	53	F	B	0		39.0	4	1+
46	1	48	F	B	0		38.5	3	1+
47	1	54	M	MF	0		38.6	2	1+
48	1	51	M	MF	0		38.8	2	1+
49	1	64	M	MF	0		38.8	2	1+
50	1	59	M	MF	0		40.0	3	1+
51	1	59	F	B	2	7	38.5	2	1+
52	1	75	M	MF	0		40.0	2	1+
53	1	16	F	MF	0		40.0	3	1+
54	1	29	M	MF	0		38.8	2	1+
55	1	11	M	MF	0		38.7	3	1+
56	1	27	M	MF	0		39.3	2	1+
57	1	43	M	B	0		38.5	2	1+
58	1	10	M	B	0		39.7	3	±
59	1	24	F	MF	3	4	39.1	2	±
60	1	61	F	MF	0		39.2	2	±
61	1	37	M	MF	0		39.6	2	±
62	1	43	F	B	0		38.6	3	±
63	1	36	M	MF	0		38.7	2	±
64	1	33	M	MF	0		39.9	2	±
65	1	24	F	B	2	7	39.2	5	±
66	1	39	F	MF	3	6	38.8	2	±
67	1	41	M	MF	0		38.0	2	±
68	1	60	F	MF	0		38.5	1	±
69	1	67	M	A	3	7	38.5	2	±
70	1	75	M	B	0		39.8	2	±
71	1	14	M	MF	3	6	40.0	1	±
72	1	74	F	MF	2	11	39.0	1	±
73	1	60	F	B	3	7	38.5	1	±
74	1	19	M	MF	3	5	38.0	1	±
75	1	20	M	B	0		38.8	2	±
76	1	16	M	A	0		39.0	3	±
77	1	31	F	MF	0		39.0	2	±
78	1	56	M	MF	0		38.6	2	±
79	1	41	M	B	0		38.4	1	−
80	1	43	M	B	0		40.00	3	−
81	1	12	F	B	3	3	37.5	1	−
82	1	21	F	MF	0		39.2	3	−
83	1	24	F	MF	0		38.8	1	−
84	1	46	M	A	3	6	39.8	2	−
85	1	26	F	MF	3	5	38.00	1	−

86	1	51	M	MF	2	7	39.6	1	−
87	1	69	M	B	0		40.0	3	−
88	1	23	F	MF	0		39.3	2	−
89	2	52	F	B	3	6	39.5	2	1+
90	2	29	M	MF	0		39.1	3	±
91	2	51	M	B	0		37.7	1	±
92	2	52	F	B	0		38.6	2	±
93	2	27	F	MF	0		38.0	2	−
94	2	34	F	MF	0		37.8	1	−
95	2	37	F	A	3	3	40.0	2	−
96	2	44	F	MF	3	5	38.0	1	−
97	2	53	F	B	0		38.6	1	−
98	2	59	F	B	3	3	37.5	2	−
99	2	59	M	B	0		38.9	1	−
100	2	55	F	MF	0		38.2	3	−
101	2	70	M	MF	0		39.2	2	−
102	2	75	F	B	0		37.5	1	−
103	2	42	M	B	0		39.6	1	−
104	2	69	M	MF	0		37.8	1	−
105	2	68	F	B	0		40.0	2	−
106	2	67	M	MF	0		40.0	2	−
107	2	75	M	A	0		37.9	3	−

Sex: F, female; M, male; A, artificial milk; B, breast milk; MF, mixed feeding.
